# Obesity in the absence of comorbidities is not related to clinically meaningful left ventricular hypertrophy

**DOI:** 10.1007/s10554-021-02207-1

**Published:** 2021-03-17

**Authors:** Andrew J. M. Lewis, Jennifer J. Rayner, Ines Abdesselam, Stefan Neubauer, Oliver J. Rider

**Affiliations:** University of Oxford Centre for Clinical Magnetic Resonance Research, University of Oxford, Oxford, OX3 9DU UK

**Keywords:** Obesity, Magnetic resonance imaging, Left ventricular hypertrophy

## Abstract

Obesity is associated with the development of left ventricular (LV) hypertrophy. Whether obesity in in the absence of comorbidities can cause LV hypertrophy to an extent which could create diagnostic uncertainty with pathological states (such as hypertrophic cardiomyopathy) is unknown. We used cine cardiovascular magnetic resonance imaging to precisely measure LV wall thickness in the septum and lateral wall in 764 people with body mass indices ranging from 18.5 kg/m^2^ to 59.2 kg/m^2^ in the absence of major comorbidities. Obesity was related to LV wall thickness across the cohort (basal septum r 0.30, P < 0.001 and basal lateral wall r 0.18, P < 0.001). Although no participant had hypertension, these associations remained highly significant after controlling for systolic blood pressure (all P < 0.01). Each 10 kg/m^2^ increase in BMI was associated with an increase in basal septal wall thickness of 1.0 mm males and 0.8 mm in females, with no statistically significant difference between genders (P = 0.1). Even in class 3 obesity (BMI > 40 kg/m^2^), no LV wall thickness > 13.4 mm in males or > 12.7 mm in females was observed in this cohort. We confirm that obesity in the absence of comorbidities is associated with LV hypertrophy, and establish that the magnitude of this change is modest even in severe obesity. LV hypertrophy > 14 mm cannot safely be attributed to obesity alone and alternative diagnoses should be considered.

## Background

Obesity is rapidly increasing in prevalence worldwide and is associated with adverse cardiovascular outcomes, including a two-fold increase in the risk of developing heart failure [1]. Obesity is also associated with cardiac remodelling in the absence of a clinical syndrome of heart failure [2], which is characterised primarily by the development of left ventricular hypertrophy (LVH). Although originally thought to be associated with mainly an eccentric pattern of left ventricular hypertrophy [3], it is now recognised that obesity can also be associated with an increase in LV wall thickness [4, 5], leading to concentric hypertrophy. The mechanisms are incompletely understood but are likely to include an increased risk of hypertension [6], pro-hypertrophic adipokine signalling [7], a hyperdynamic haemodynamic state [8] and others.

One important clinical question arising from these observations is the extent to which LVH can be confidently attributed to obesity in the absence of comorbidities. Current diagnostic imaging criteria for the diagnosis of hypertrophic cardiomyopathy include a maximum wall thickness of > 13–15 mm [9, 10], especially in the context of an appropriate family history. International guidelines however also stipulate that wall thickness should be interpreted in the context of a history of other factors such as hypertension or athletic training [11], as it is recognised that these conditions can be associated with LV hypertrophy which can in some cases be > 15 mm and hence cause diagnostic uncertainty with pathological states such as hypertrophic cardiomyopathy.

Previous studies of LV remodelling in obesity have evaluated the effects of obesity upon LV mass and volume, whereas clinical evaluation and diagnostic criteria for suspected cardiomyopathy instead rely upon maximum wall thickness as the key measure of interest. Specifically, whether obesity in isolation could cause LVH to an extent which could otherwise create clinical suspicion for (and diagnostic uncertainty with) pathological states including hypertrophic cardiomyopathy (i.e. a wall thickness of > 15 mm) is unknown. An additional reason for this uncertainty is the difficulty in obtaining precise measures of LV wall thickness and in visualising the entire LV in people with obesity using transthoracic echocardiography, where acoustic windows are frequently suboptimal.

In order to address this question and understand the extent to which obesity could cause LVH in the absence of comorbidities, we used cardiovascular magnetic resonance (CMR) imaging to precisely measure LV wall thickness at the basal septum and basal lateral wall in people with no known or suspected cardiovascular disease.

## Materials and methods

**Fig. 1:**
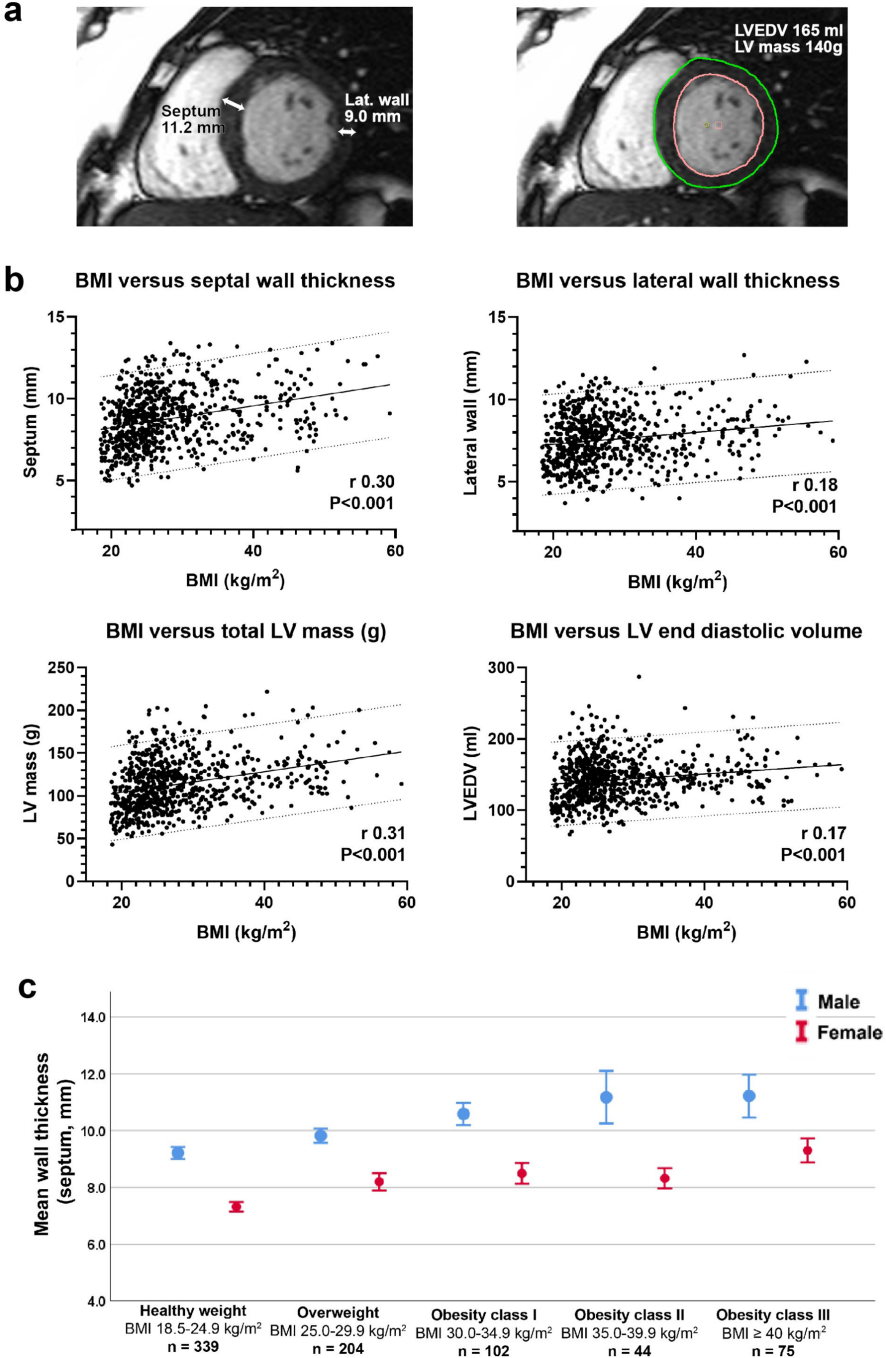
Panel **a** precise measurement of LV wall thickness at the septum and lateral wall as well as LV mass and LV end-diastolic volume using CMR despite class III obesity with a BMI 47.3 kg/m^2^. Panel **b** obesity is related to wall thickness in both the basal septum and basal lateral wall. Obesity is also related to an increase in total LV mass and LV end diastolic volume. Panel **c** in the absence of comorbidities even severe obesity is not associated with LV hypertrophy of > 13.4 mm in males or > 12.7 mm in females

We used cardiovascular magnetic resonance (CMR) imaging to precisely measure LV wall thickness at the basal septum and basal lateral wall (Fig. 1a) in 764 people with no known or suspected cardiovascular disease. These participants had attended for research studies at our institution (e.g. as control participants or to studies specifically investigating the effects of increasing body weight). We did not include participants referred for clinical evaluation of known or suspected cardiovascular disease in order to isolate the effects of increasing BMI.

CMR imaging was performed using Siemens MR systems at field strengths 1.5–3 T at the University of Oxford Centre for Clinical Magnetic Resonance Research. Cine imaging was conducted using standard manufacturer sequences with retrospective electrocardiographic gating.

Inclusion criteria for this study included an age range between 18 and 80 years of age and the ability to provide written informed consent to participate in a research study. Exclusion criteria were an inability to provide informed consent, a history of suspected or known cardiovascular disease, a history of hypertension (systolic blood pressure > 140 mmHg, diastolic blood pressure > 90 mmHg), competitive athletic training, a BMI below 18.5 kg/m^2^, a history of diabetes requiring medication treatment, or any safety-based contraindication to an MRI examination such as a non-conditional implantable electronic cardiac device.

Image analysis was conducted using cvi42 software (Circle, Calgary) by two experienced CMR cardiologists. LV wall thickness was measured by placing callipers across the septum and lateral walls at the point of maximum wall thickness respectively (Fig. 1a). LV mass and LV end-diastolic volume were derived by placing LV endocardial and epicardial contours covering the LV in end diastole. Statistical analysis was performed using SPSS and GraphPad Prism software. Data were evaluated for normality of distribution and are presented as median (IQR) or mean (SD) as appropriate with linear regression performed according to standard techniques.

## Results

Baseline characteristics are provided in Table 1. The study participants had a median age of 36 (IQR 26–48) years (range 19–80 years) and were normotensive (median systolic blood pressure (BP) 119 (112–128) mmHg, diastolic BP 73 (68–79) mmHg) and normocholesterolaemic (median fasting total cholesterol 4.8 (IQR 4.1–5.4) mmol/l). No participant had a diagnosis of diabetes requiring treatment and the median fasting blood glucose across the cohort was 4.9 ± 0.57 mmol/L (range 3.3–6.9).

**Table 1:** Baseline characteristics

Age, median (IQR), years	36 (26–48)
Female sex, n, (%)	433 (57%)
Weight, median (IQR), kg	82 (66–91)
Height, median (IQR), m	1.71 (1.64–1.79)
WHO BMI category [kg/m^2^, n (%)]	
18.5–24.9 kg/m^2^	339 (44%)
25.0–29.9 kg/m^2^	204 (27%)
30.0–34.9 kg/m^2^	102 (13%)
35.0–39.9 kg/m^2^	44 (6%)
Above 40 kg/m^2^	75 (10%)
Systolic blood pressure, median (IQR), mmHg	119 (112–128)
Diastolic blood pressure, median (IQR), mmHg	73 (68–79)
Serum glucose, median (IQR), mmol/L	4.8 (4.5–5.2)
Total cholesterol, median (IQR)	4.8 (4.1–5.4)
LVEDV median, median (IQR), ml	141 (121–161)
LV mass median, median (IQR), g	111 g (91–132)
LVEF, mean (SD), %	67 (6.2)

The study participants covered a wide range of body mass indices (cohort BMI range 18.5–59.2 kg/m^2^) and participants with obesity were well represented. Of the 762 participants, 339 (44%) were of normal weight, whilst 204 (27%) were overweight (BMI 25.0–29.9 kg/m^2^), and 221 (29%) were obese (BMI > 30 kg/m^2^). Of the participants with obesity, 102 (13%) had class I obesity, 44 (6%) had class II obesity and 75 (10% had class III obesity.

We found that obesity was related to LV wall thickness across the cohort (basal septum r 0.30, P < 0.001 and basal lateral wall r 0.18, P < 0.001 (Fig. 1b and Table 2). Although no participant had hypertension (systolic BP < 140 mmHg, diastolic BP < 90 mmHg), these associations remained highly statistically significant (P < 0.01) after controlling for differences in systolic blood pressure occurring within the normal range. BMI was as expected also related to total LV mass (r 0.31, P < 0.001) and to a lesser extent to LV end diastolic volume (r 0.17, P < 0.001) consistent with prior data.

**Table 2:** Breakdown of LV wall thickness at the septum and lateral wall and total LV mass according to WHO BMI categories

WHO BMI category	Septal diameter (mm ± SD)	Lateral wall diameter (mm ± SD)	LV mass (g ± SD)
Healthy weight (18.5–24.9 kg/m^2^)	8.2 ± 1.6	7.3 ± 1.5	106 ± 29
Overweight (25.0–29.9 kg/m^2^)	9.1 ± 1.6	7.8 ± 1.6	117 ± 28
Obesity class I (30.0–34.9 kg/m^2^)	9.3 ± 1.7	7.7 ± 1.5	118 ± 28
Obesity class II (35.0–39.9 kg/m^2^)	8.9 ± 1.5	7.4 ± 1.6	121 ± 28
Obesity class III (≥ 40 kg/m^2^)	9.7 ± 1.8	8.2 ± 1.5	134 ± 28

Mean ± SD

The association between BMI and wall thickness was present in both males (septum r 0.42, P < 0.001, lateral wall r 0.21, P < 0.001) and females (septum r 0.45, P < 0.001, lateral r 0.31, P < 0.001). When comparing LV wall thickness in people of healthy weight (BMI 18.5–24.9 kg/m^2^) versus those with class III obesity (BMI > 40 kg/m^2^), septal wall thickness increased from 9.2 ± 1.3 mm to 11.2 ± 1.5 mm in males, and from 7.3 ± 1.2 mm to 9.3 ± 1.6 mm in females (Fig. 1c; all P < 0.001).

Using linear regression, we found that every 10 kg/m^2^ increase in BMI was associated with an increase in basal septal wall thickness of 1.0 mm males and 0.8 mm in females. There was no statistically significant difference in the rate of increase in LV wall thickness with increasing body weight between genders (P = 0.1). Even in the most severe class 3 obesity (with a BMI > 40 kg/m^2^), in the absence of comorbidities, no LV wall thickness > 13.4 mm in males or > 12.7 mm in females was observed in this cohort (Fig. 1c).

## Discussion

Our findings confirm that obesity is related to an increase in LV wall thickness, particularly in the basal septum, and add that the magnitude of this change is modest in the absence of comorbidities. These findings establish that, unlike hypertension or athletic training, even severe obesity in isolation does not cause LVH to a degree which would be sufficient to cause diagnostic uncertainty with pathological hypertrophic states such as hypertrophic cardiomyopathy.

The mechanisms behind the development of LVH in obesity remain incompletely understood. They are however likely to be multifactorial and to include haemodynamic effects via an increase in total blood volume/cardiac whilst concentric remodelling might also reflect activation of myocardial hypertrophic signalling pathways via leptin and another adipokines [7, 12, 13]. Obesity is known to be associated with hypertension, and LVH in the absence of hypertension [14], though participants with hypertension were no included in this study.

Previous studies have highlighted a degree of gender dependence of the LV hypertrophic response to obesity, with males demonstrating predominantly concentric hypertrophy, and females demonstrates a mixed eccentric/concentric phenotype [4]. In this study, the degree of wall thickness tended to be higher in the men than women, though the rate of increase in LV wall thickness with increasing BMI was not different between genders and in neither did LV wall thickness exceed 15 mm, even in class III obesity. The association between BMI and an increase in both total LV mass and LV end-diastolic volume in this study are both consistent with prior work and the ability of CMR to provide whole heart imaging to derive LV mass is a strength of the technique.

LVH associated with obesity can regress following dietary or surgical weight loss [15]. The relationship between body weight and the risk of heart failure is complicated, with obesity associated to an increased risk of developing heart failure but an apparently paradoxical protective effect once heart failure is established [16–18]. Obesity is also independently associated with an increased in LV mass and the risk of developing heart failure in hypertrophic cardiomyopathy [19]. Randomised trials are required to determine the optimal strategy if any for body weight management in heart failure and cardiomyopathy.

One limitation of this study is that gadolinium was not routinely administered, and late gadolinium imaging was therefore not performed. Late gadolinium imaging has an important role in the evaluation of suspected cardiomyopathy, especially when wall thickness is increased. Likewise, native T1 mapping, which can be abnormal in specific forms of cardiomyopathy including Anderson–Fabry disease and haemochromatosis, was not performed in this study. However maximal LV wall thickness remains the primary imaging diagnostic criterion for cardiomyopathy in international guidelines. As expected the strength of the correlations in this study were moderate, as other factors affect LV wall thickness.

The findings from this study confirm that obesity per se in the absence of comorbidities is related to an increase in LV wall thickness, particularly in the basal septum, but importantly establish that the magnitude of this hypertrophy is modest even in severe obesity. We conclude that a basal septal wall thickness of > 14 mm in men and > 13 mm in women therefore cannot be confidently attributed to obesity alone and alternative diagnoses should be considered in this situation.

## Data Availability

The study data supporting the manuscript are available from the corresponding author upon approval of a reasonable request.

## References

[1] Kenchaiah S, Evans JC, Levy D (2002). Obesity and the risk of heart failure. N Engl J Med.

[2] Abel ED, Litwin SE, Sweeney G (2008). Cardiac remodeling in obesity. Physiol Rev.

[3] Chakko S, Mayor M, Allison MD (1991). Abnormal left ventricular diastolic filling in eccentric left ventricular hypertrophy of obesity. Am J Cardiol.

[4] Rider OJ, Lewandowski A, Nethononda R (2013). Gender-specific differences in left ventricular remodelling in obesity: insights from cardiovascular magnetic resonance imaging. Eur Heart J.

[5] Woodiwiss AJ, Libhaber CD, Majane OH (2008). Obesity promotes left ventricular concentric rather than eccentric geometric remodeling and hypertrophy independent of blood pressure. Am J Hypertens.

[6] Hall JE (2000). Pathophysiology of obesity hypertension. CurrHypertens Rep.

[7] Sader S, Nian M, Liu P (2003). Leptin: a novel link between obesity, diabetes, cardiovascular risk, and ventricular hypertrophy. Am Heart Assoc.

[8] Vasan R (2003). Cardiac function and obesity.

[9] Elliott PM, Anastasakis A, Members ATF (2014). 2014 ESC guidelines on diagnosis and management of hypertrophic cardiomyopathy: the task force for the diagnosis and management of hypertrophic cardiomyopathy of the European society of cardiology (ESC). Eur Heart J.

[10] Ommen SR, Mital S, Burke MA (2020). 2020 AHA/ACC guideline for the diagnosis and treatment of patients with hypertrophic cardiomyopathy: a report of the American College of Cardiology/American Heart Association joint committee on clinical practice guidelines. J Am CollCardiol.

[11] Rawlins J, Bhan A, Sharma S (2009). Left ventricular hypertrophy in athletes. Eur J Echocardiogr.

[12] Allison MA, Bluemke DA, McClelland R (2013). Relation of leptin to left ventricular hypertrophy (from the multi-ethnic study of atherosclerosis). Am J Cardiol.

[13] Park M, Sweeney G (2013). Direct effects of adipokines on the heart: focus on adiponectin. Heart Fail Rev.

[14] Woodiwiss AJ, Norton GR (2015). Obesity and left ventricular hypertrophy: the hypertension connection. CurrHypertens Rep.

[15] Rider OJ, Francis JM, Ali MK (2009). Beneficial cardiovascular effects of bariatric surgical and dietary weight loss in obesity. J Am CollCardiol.

[16] Ponikowski P, Voors AA, Anker SD (2016). 2016 ESC Guidelines for the diagnosis and treatment of acute and chronic heart failure: the task force for the diagnosis and treatment of acute and chronic heart failure of the European society of cardiology (ESC) developed with the special contribution of the heart failure association (HFA) of the ESC. Eur Heart J.

[17] Rayner J, Neubauer S, Rider O (2015). The paradox of obesity cardiomyopathy and the potential for weight loss as a therapy. Obes Rev.

[18] Lavie CJ, Alpert MA, Arena R (2013). Impact of obesity and the obesity paradox on prevalence and prognosis in heart failure. JACC Heart Fail.

[19] Olivotto I, Maron BJ, Tomberli B (2013). Obesity and its association to phenotype and clinical course in hypertrophic cardiomyopathy. J Am CollCardiol.

